# Bat Caliciviruses and Human Noroviruses Are Antigenically Similar and Have Overlapping Histo-Blood Group Antigen Binding Profiles

**DOI:** 10.1128/mBio.00869-18

**Published:** 2018-05-22

**Authors:** Jacob F. Kocher, Lisa C. Lindesmith, Kari Debbink, Anne Beall, Michael L. Mallory, Boyd L. Yount, Rachel L. Graham, Jeremy Huynh, J. Edward Gates, Eric F. Donaldson, Ralph S. Baric

**Affiliations:** a Department of Epidemiology, the University of North Carolina at Chapel Hill, Chapel Hill, North Carolina, USA; b Department of Natural Sciences, Bowie State University, Bowie, Maryland, USA; c Department of Microbiology and Immunology, the University of North Carolina at Chapel Hill, Chapel Hill, North Carolina, USA; d University of Maryland Center for Environmental Science, Frostburg, Maryland, USA; Mailman School of Public Health, Columbia University

**Keywords:** calicivirus, histo-blood group antigens, noroviruses, sialic acid, zoonotic infections

## Abstract

Emerging zoonotic viral diseases remain a challenge to global public health. Recent surveillance studies have implicated bats as potential reservoirs for a number of viral pathogens, including coronaviruses and Ebola viruses. *Caliciviridae* represent a major viral family contributing to emerging diseases in both human and animal populations and have been recently identified in bats. In this study, we blended metagenomics, phylogenetics, homology modeling, and *in vitro* assays to characterize two novel bat calicivirus (BtCalV) capsid sequences, corresponding to strain BtCalV/A10/USA/2009, identified in Perimyotis subflavus near Little Orleans, MD, and bat norovirus. We observed that bat norovirus formed virus-like particles and had epitopes and receptor-binding patterns similar to those of human noroviruses. To determine whether these observations stretch across multiple bat caliciviruses, we characterized a novel bat calicivirus, BtCalV/A10/USA/2009. Phylogenetic analysis revealed that BtCalV/A10/USA/2009 likely represents a novel *Caliciviridae* genus and is most closely related to "recoviruses." Homology modeling revealed that the capsid sequences of BtCalV/A10/USA/2009 and bat norovirus resembled human norovirus capsid sequences and retained host ligand binding within the receptor-binding domains similar to that seen with human noroviruses. Both caliciviruses bound histo-blood group antigens in patterns that overlapped those seen with human and animal noroviruses. Taken together, our results indicate the potential for bat caliciviruses to bind histo-blood group antigens and overcome a significant barrier to cross-species transmission. Additionally, we have shown that bat norovirus maintains antigenic epitopes similar to those seen with human noroviruses, providing further evidence of evolutionary descent. Our results reiterate the importance of surveillance of wild-animal populations, especially of bats, for novel viral pathogens.

## IMPORTANCE

Caliciviruses are rapidly evolving viruses that cause pandemic outbreaks associated with significant morbidity and mortality globally. The animal reservoirs for human caliciviruses are unknown; bats represent critical reservoir species for several emerging and zoonotic diseases. Recent reports have identified several bat caliciviruses but have not characterized biological functions associated with disease risk, including their potential emergence in other mammalian populations. In this report, we identified a novel bat calicivirus that is most closely related to nonhuman primate caliciviruses. Using this new bat calicivirus and a second norovirus-like bat calicivirus capsid gene sequence, we generated virus-like particles that have host carbohydrate ligand binding patterns similar to those of human and animal noroviruses and that share antigens with human noroviruses. The similarities to human noroviruses with respect to binding patterns and antigenic epitopes illustrate the potential for bat caliciviruses to emerge in other species and the importance of pathogen surveillance in wild-animal populations.

## INTRODUCTION

Zoonotic diseases remain among the greatest overall threats to global public health. Current estimates indicate that 70% of all new emerging infectious diseases are of zoonotic origin (1, 2). Historical examples of zoonotic pathogens include avian and swine influenza viruses, henipaviruses, and severe acute respiratory syndrome coronavirus (SARS-CoV). More recently, the emergence of Ebola virus and of Middle East respiratory syndrome coronavirus (MERS-CoV) has resulted in local and intercontinental pandemics, with animal reservoirs believed to play a critical role in their emergence (3–6). Bats, which are among the most common reservoirs for these zoonotic pathogens, have been identified as reservoirs or potential reservoirs for a number of highly pathogenic viruses, including SARS-CoV (7–9), MERS-CoV (10), and Ebola virus (4). A recent study of the viral diversity within Pteropus giganteus identified 55 viruses spanning nine viral families. The authors extrapolated these findings to estimate that a minimum of 320,000 unknown viruses are currently circulating among wild-mammal populations globally (11). Nevertheless, the prevalence of viruses in wild mammals, particularly among bats, remains grossly understudied. Thus, the surveillance of local and global bat populations can help identify potentially zoonotic or pandemic pathogens prior to their emergence.

The *Caliciviridae* family consists of nonenveloped, positive-sense RNA viruses subclassified into five genera, *Vesivirus*, *Lagovirus*, *Norovirus*, *Sapovirus*, and *Nebovirus*, along with two unclassified genera, "*Recovirus*" and "*Valovirus*." Caliciviruses infect a wide range of hosts, including humans and wildlife and domestic, companion, and agricultural animals, although the origins and radiation of these viruses through mammalian populations remain uncertain. For instance, the *Norovirus* genus, which contains the most common and well-known caliciviruses, consists of six genogroups and has an expansive host range. Primarily identified in humans, noroviruses (NoVs) have also been identified in canine (12–14), feline (14, 15), swine (16, 17), murine (18, 19), ovine (20), and bovine (21) species. Other caliciviruses have been identified in sea lions (22–24), minks (25), rabbits (26, 27), chickens (28), geese (29, 30), fish (31), and nonhuman primates (32, 33), indicating the broad host range of the *Caliciviridae*.

The mechanisms by which the caliciviruses have expanded their host range and emerged to infect the human population are currently unknown. Some human noroviruses (HuNoVs), the most well-known and prevalent caliciviruses, undergo epochal evolution, with a new pandemic strain emerging every 2 to 5 years (34); new strains emerge following herd immunity-induced evolution within antigenic and receptor binding epitopes (35). The immunocompromised human population might also serve as a reservoir from which pandemic noroviruses might emerge, while zoonotic transmission remains likely but unsubstantiated (36–39). However, antibodies against animal caliciviruses have been detected in the human population, suggesting the potential for cross-species transmission events, though clinical disease has yet to be confirmed (40–44). Thus, the identification and study of animal caliciviruses, including bat caliciviruses (BtCalVs), and their potential role in zoonotic disease and cross-species transmission potential represent significant gaps in global health preparedness.

Several recent reports have identified *Caliciviridae* sequences resident within several different bat species worldwide (45–50). Phylogenetic analyses have revealed that these viruses are closely related to sapoviruses (45, 46, 49) and "valoviruses" (46). More importantly, bat noroviruses have been reported in two separate studies of microbats in China (50, 51), including one nearly full-length *Norovirus* genome (here referred to as bat norovirus [BtNoV]) (50). However, in the absence of any biological data, the potential of these viruses to transmit into other mammals, including humans, is difficult to predict from genome-length sequences. These reports of novel caliciviruses identified in bats not only stress the need for surveillance but also emphasize the need for detailed biological and immunologic characterization of new caliciviruses identified in wild-animal populations, particularly bats, to provide potential insights into cross-species transmission potential and human health.

Here, we evaluate the antigenicity and receptor-binding profiles of two bat caliciviruses. Specifically, we generated virus-like particles (VLPs) from the BtNoV capsid sequence (50) that were detected with HuNoV-derived hyperimmune serums, indicating antigenic relationships shared between HuNoVs and BtNoV. Further, we characterize a novel bat calicivirus capsid sequence isolated from Perimyotis subflavus, the tri-colored bat, in the Mid-Atlantic region of the United States (52). Phylogenetic analyses revealed that this bat capsid sequence likely represents a novel calicivirus that is most closely related to non-human caliciviruses, such as lagoviruses and "recoviruses." We used VLPs from the newly identified bat calicivirus and BtNoV to assess their potential carbohydrate patterns in comparison with those of human noroviruses. Here, we describe host carbohydrate ligand-receptor binding patterns that overlapped between bat and human caliciviruses, suggesting that bat caliciviruses have the potential to clear one barrier to cross-species movement. Our data also suggest that bat caliciviruses share antigenic epitopes with HuNoVs, indicating a potential linkage via evolutionary descent.

## RESULTS

### BtNoV is antigenically similar to HuNoVs.

**FIG 1:**
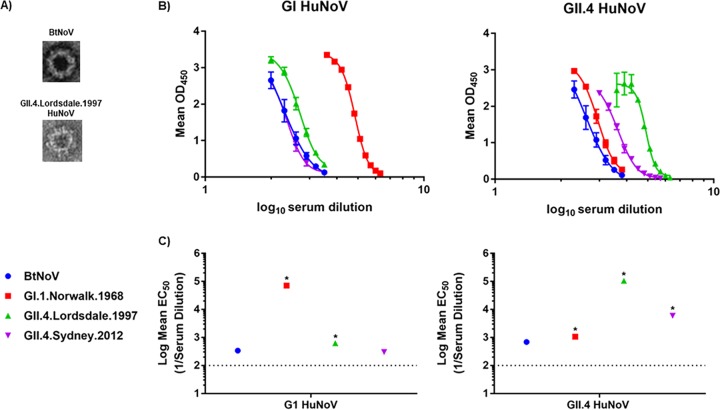
BtNoV VLPs share antigenic epitopes with HuNoVs. (A) We generated VLPs for BtNoV using the VEE replicon in BHK cells. BtNoV VLPs had sizes and morphologies similar to those of GII.4.Lordsdale.1997 HuNoV VLPs. (B and C) GI (left panels) and GII.4 (right panels) HuNoV hyperimmune serums were used to determine antigenic relationships between BtNoV and HuNoV (GI.1.Norwalk.1968, GII.4.Lordsdale.1997, GII.4.Sydney.2012) VLPs. (B) Data presented are the mean absorbance values (optical density at 450 nm [OD_450_]) measured following subtraction of negative-control absorbance values ± SEM for GI serum and GII.4 serum. (C) Mean EC_50_ ± SEM (1/serum dilution) for reactivity of each antibody with each VLP. Each panel is representative of results from two independent experiments conducted in duplicate. *, VLP with EC_50_ value significantly different from EC_50_ of BtNoV (one-way ANOVA, *P* < 0.05).

We first sought to identify if BtNoV maintained any antigenic similarity with noroviruses, specifically HuNoVs. Thus, we produced BtNoV VLPs using alphavirus replicon vectors in BHK cells. The BtNoV capsid sequence formed VLPs of approximately 40 nm in diameter that resembled VLPs generated from genotype II 4.1997.Lordsdale (GII.4.1997.Lordsdale) HuNoV (Fig. 1A). To discern the potential antigenic relationships of BtNoV with HuNoVs, we utilized an enzyme immunoassay (EIA) (53) with hyperimmune serums against GI or GII.4 HuNoVs and against VLPs from BtNoV and a panel of GI and GII.4 HuNoVs (Fig. 1B and C).

As anticipated, hyperimmune GI and GII.4 HuNoV-derived serums strongly reacted with their homotypic VLPs (Fig. 1B). Specifically, GI.1.Norwalk.1968 HuNoV VLPs reacted strongly with the homotypic GI HuNoV hyperimmune serums (Fig. 1C) (50% effective concentration [EC_50_] of 1/71,456); similarly, both GII.4.Lordsdale.1997 and GII.4.Sydney.2012 HuNoV VLPs reacted strongly with the homotypic GII.4 HuNoV hyperimmune serums (EC_50_s of 1/104,962 and 1/5,984, respectively) (Fig. 1C). Interestingly, BtNoV VLPs reacted with both GI and GII.4 serums similarly to the VLPs derived from heterotypic HuNoVs (Fig. 1B). The BtNoV VLPs required significantly higher levels of GI (EC_50_ of 1/340.4) and GII.4 (EC_50_ of 1/694.4) serums than the homotypic VLPs for detection by EIA. It is noteworthy that the BtNoV and GII.4.Sydney.2012 VLPs had almost identical EC_50_s for binding GI HuNoV-derived serums.

### BtNoV binds histo-blood group antigens similarly to a historic HuNoV.

**FIG 2:**
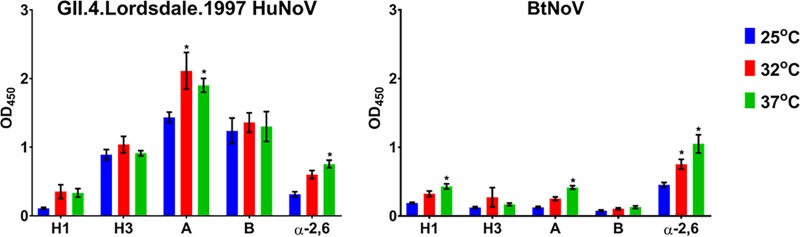
BtNoV VLPs bind to HBGAs in a temperature-dependent manner similar to that seen with GII.4.Lordsdale.1997 HuNoV VLPs. We evaluated interactions between GII.4.Lordsdale.1997 HuNoV VLPs (left) and BtNoV VLPs (right) against Hs1, Hs3, A, and B HBGAs and α-2,6 sialic acids under a gradient of physiological conditions. Data presented are mean OD_450_ absorbance values following subtraction of negative-control absorbance ± SEM. Each VLP-HBGA interaction was evaluated in duplicate in two independent experiments. *, VLP-HBGA at 32°C and 37°C with a mean OD_450_ absorbance value significantly different from the mean absorbance value at 25°C (two-way ANOVA, *P* ≤ 0.05).

Since we had established that BtNoV VLPs are antigenically similar to HuNoV-derived VLPs and since host receptor ligand differences are among the primary barriers to calicivirus cross-species transmission, we next sought to identify whether BtNoV VLPs could bind histo-blood group antigens (HBGAs) *in vitro*. Additionally, as bats are heterothermic, their body temperatures fluctuate from ambient temperature during torpor to 37°C during their active state (54, 55). Further, temperature changes have been shown to induce conformational changes in the HuNoV capsid, altering the presentation of antigenic epitopes (56). Thus, we hypothesized that temperature changes would impact these potential VLP-carbohydrate ligand binding interactions (Fig. 2). To address these issues, we utilized an *in vitro* synthetic HBGA carbohydrate binding assay and incubated the BtNoV VLPs in comparison with GII.4.Lordsdale.1997 HuNoV VLPs at a gradient of temperatures that capture a range of physiological temperatures identified in bats: 25°C, 32°C, and 37°C.

As we hypothesized, BtNoV VLPs bound to HBGAs and their carbohydrate binding interactions were impacted by temperature in a manner similar to that seen with HuNoVs (Fig. 2). Specifically, GII.4.Lordsdale.1997 VLPs bound significantly better to A type HBGA at 32°C and 37°C and α-2,6-sialic acid at 37°C. A similar trend, though the data were not statistically significant, was observed with these VLPs with respect to H type 1 HBGAs. Similarly, the BtNoV VLPs bound significantly better to H type 1 and A at 37°C and α-2,6-sialic acid at 32°C and 37°C. We noted no trends with respect to correlations between incubation temperature and the binding of either VLP to H type 3 or B HBGAs, though BtNoV VLPs did not bind to these HBGAs as well as HuNoV-derived VLPs. These results indicate that the temperature may induce conformational changes within the BtNoV capsid similarly to HuNoVs (56), that the transmission of these bat viruses may depend on the activity level and body temperature of bats, and that bats may carry or transmit HBGA-binding viruses under a gradient of physiological conditions.

### The A10 bat calicivirus is phylogenetically related to "recoviruses."

**FIG 3:**
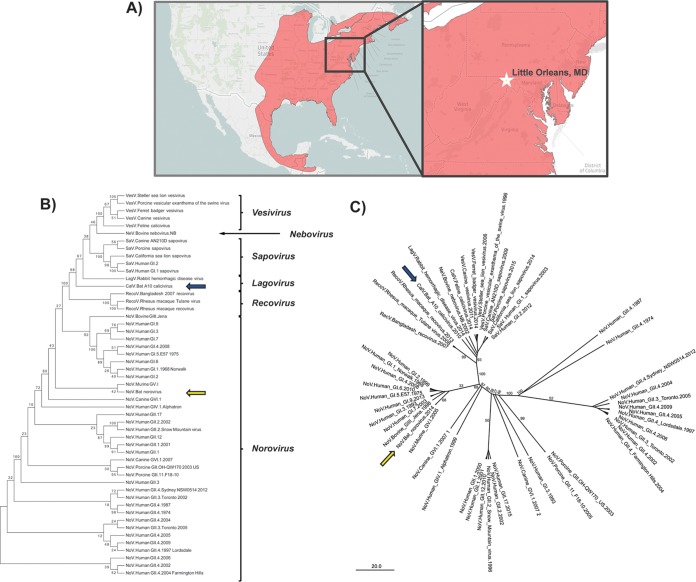
Identification of a novel North American bat viral sequence that is phylogenetically related to *Caliciviridae*. We identified a sequence from Perimyotis subflavus that was related to *Caliciviridae* capsid sequences. (A) The distribution of P. subflavus throughout North America and Central America is marked in red. The sampling site of Little Orleans, MD, is marked by a star (inset). The map was generated using data from the International Union for Conservation of Nature (IUCN) with permission. (B and C) A maximum-likelihood cladogram (B) and radial phylograms (C) of 51 known calicivirus S domain amino acid sequences were generated based on the JTT matrix-based amino acid substitution model (SB) and the Jukes-Cantor genetic distance model (SC). BtNoV and BtCalV/A10 are indicated by yellow and blue arrows, respectively.

We next hypothesized that other caliciviruses are circulating in bats and, similarly to our observations with BtNoV, would be capable of binding HBGAs *in vitro*. In a previous report, we identified multiple viral sequences from *Coronaviridae*, *Herpesviridae*, *Iflaviridae* insect viruses, *Tymoviridae* plant viruses, and *Podoviridae* bacteriophage in several species of bats near an abandoned railway tunnel (Indigo Tunnel) in Allegany County, MD, USA, approximately 1 mi east of Little Orleans, MD (Fig. 3A) (52). Additionally, we assembled two contigs of 865 nucleotides and 534 nucleotides from 232 and 3 sequence reads from the guano of Perimyotis subflavus, the tri-colored bat. A BLAST search showed that the assembled contigs most closely matched the capsid sequences of GII HuNoVs and feline calicivirus, indicating the identification of a 510-amino-acid capsid sequence (GenBank accession number MH259583) of a bat calicivirus, BtCalV/A10/USA/2009 (here referred to as A10 bat calicivirus [BtCalV/A10]). It is noteworthy that we did not identify similar calicivirus sequences from the other sampled bat species.

To identify how BtCalV/A10 and BtNoV are related to known *Caliciviridae* capsid sequences, we analyzed the amino acid sequences of the conserved shell (S) domain of the capsid protein due to the extensive variability within the protruding domain of the VP1 capsid protein (57, 58). We generated S domain phylogenetic trees and cladograms from 51 known calicivirus S domain sequences (Fig. 3B and C). First, we noted that BtNoV and BtCalV/A10 clustered in separate clades. BtCalV/A10 was directly rooted by the nonhuman primate "recoviruses" and further rooted a clade containing lagoviruses, human and animal sapoviruses, vesiviruses, and neboviruses. Based on this analysis, BtCalV/A10 is most closely related to rabbit hemorrhagic disease virus (RHDV) and the "recoviruses" but likely belongs to a novel genus within *Caliciviridae*. Conversely, BtNoV clustered with murine norovirus (MNV) and rooted a clade of viruses that included BtCalV/A10, the animal caliciviruses, and GI and GIII noroviruses. While BtCalV/A10 clustered most closely with RHDV and next most closely with the "recoviruses," it maintained only 22% sequence identity and 19% to 22% sequence identity with those viruses, respectively (see Table S1 in the supplemental material). Conversely, BtNoV maintained 60% amino acid sequence identity with its closest relative, MNV, and only 23% similarity with BtCalV/A10. Similar phylogenetic relationships were observed in VP1 analysis (see Fig. S1 in the supplemental material). Taken together, our phylogenetic results suggest that BtCalV/A10 most likely represents a genus and species distinct from all of the previously identified caliciviruses; in contrast, based on accepted norovirus classifications (59), BtNoV likely belongs to genogroup V (GV) with MNV.

10.1128/mBio.00869-18.1 FIG S1  Phylogenetic analysis of BtCalV/A10 and BtNoV. A maximum-likelihood cladogram (A) and radial phylograms (B) of 50 known calicivirus VP1 capsid amino acid sequences were generated based on the JTT matrix-based amino acid substitution model (SA) and the Jukes-Cantor genetic distance model (SB). BtNoV and BtCalV/A10 are indicated by yellow and blue arrows, respectively. Download FIG S1, TIF file, 0.2 MB. Copyright © 2018 Kocher et al. 2018 Kocher et al. This content is distributed under the terms of the Creative Commons Attribution 4.0 International license.

10.1128/mBio.00869-18.2 TABLE S1  Sequence similarity between BtCalV/A10 VP1 and S domain amino acid sequences relative to other caliciviruses. The BtCalV/A10 VP1 and S domain sequences were aligned in VectorNTI, and the data are presented as percent consensus amino acid and sequence identity. Download TABLE S1, DOCX file, 0.01 MB. Copyright © 2018 Kocher et al. 2018 Kocher et al. This content is distributed under the terms of the Creative Commons Attribution 4.0 International license.

### Homology modeling of BtCalV/A10 and BtNoV using HuNoVs and MNV.

Previous works have solved the capsid and P domain structures of several caliciviruses (60, 61). To determine how the BtCalV/A10 and BtNoV capsids structurally compare to known human and animal norovirus capsids, we utilized predictive structural modeling to illustrate the full-length BtCalV/A10 and BtNoV capsid structures. First, we used PHYRE2 open source software to determine if BtCalV/A10 contained known calicivirus capsid structures followed by structural homology modeling using Modeller software from the Max Planck Institute Bioinformatics Toolkit (62–64). Due to its low amino acid similarity to known calicivirus sequences, we also modeled the BtCalV/A10 sequence using HuNoVs (PDB identifiers [IDs]: 2OBT and 4WZT) and MNV (PDB ID: 3LQE) as backbones to capture multiple potential structures and predict potential sites of HBGA binding (see Fig. 5).

**FIG 4:**
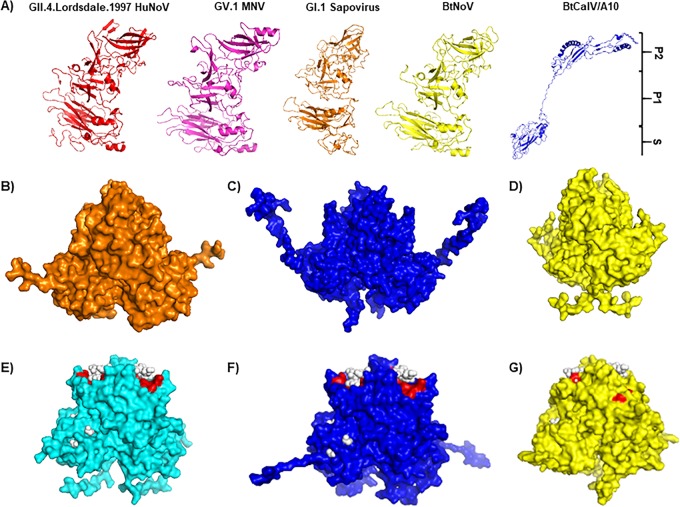
Bat caliciviruses retain host ligand binding sites similar to those of HuNoVs. (A) Modeling of full-length VP1 capsid sequence from HuNoV, MNV, GI.1 human sapovirus, BtNoV, and BtCalV/A10 using the PHYRE2 Protein Recognition Server. MNV (B) and GII.4 HuNoVs (E) were used as backbones for predictive homology modeling with Modeller software from the Max Planck Institute. The P dimers of BtCalV/A10 were modeled from MNV (C) and GII.4 HuNoVs (F). The P dimers of BtNoV were modeled from MNV (D) and GII.4 HuNoVs (G). In panels E to G, ligand binding sites were predicted by the 3DLigandSite and are marked in red. (E to G) Type A trisaccharide HBGA is indicated by white spheres.

As expected, the PHYRE2 software predicted the structure of several calicivirus full-length capsids, including GII.4.1997.Lordsdale HuNoV, MNV, GI.1 human sapovirus, and BtNoV (Fig. 4A), supporting the use of PHYRE2 software for predictive homology modeling. These models folded into distinct structures similar to previously reported calicivirus structures (Fig. 4A), including β-sheets in the S domain and P2 subdomains (46). Conversely, PHYRE2 determined that the BtCalV/A10 capsid formed distinct S and P2 subdomains similar to those of other caliciviruses but contained extensive disorder and unpredictability within the P1 subdomain, which corresponds with the capsid sequence size of BtCalV/A10 being smaller than that seen with other caliciviruses. While the predicted BtCalV/A10 structure contained β-sheets in the S domain similar to those seen with the other calicivirus structures (Fig. 4A), we also observed several α-helices within the P2 subdomain rather than the β-sheets observed in known calicivirus structures. The predictive modeling by PHYRE2 suggested two conclusions: the presence of β-sheets in the S domain suggested that the BtCalV/A10 capsid sequence resembled a calicivirus capsid sequence, but the unrecognized structures in P1 and α-helices in P2 suggested that BtCalV/A10 contained novel structures dissimilar from the structures in other caliciviruses, further supporting its tentative placement as a novel genus within *Caliciviridae*.

As the PHYRE2 modeling software did not fully predict the structure of the BtCalV/A10 capsid protein, we next modeled the BtCalV/A10 and BtNoV P dimers from the most closely related animal norovirus, MNV (Fig. 4B to D). Alignment of the P domains revealed that the BtCalV/A10 sequence had four insertions within the P domain compared to the MNV P domain template; similarly, the BtNoV sequence contained three insertions and three deletions compared to MNV (data not shown). However, as expected, the homologous models for both BtCalV/A10 (Fig. 4C) and BtNoV (Fig. 4D) resembled the MNV P domain backbone (Fig. 4B).

We also modeled the BtCalV/A10 and BtNoV P dimers on the backbone of two GII.4 HuNoVs (Fig. 4E to G). Compared to the HuNoV sequences, the BtCalV/A10 P domain sequence had six insertions and four deletions, while the BtNoV P domain sequence had three insertions and five deletions (data not shown). These modifications did not affect modeling of the P dimers from HuNoVs for either of the bat virus sequences. The BtCalV/A10 dimer (Fig. 4F) and BtNoV dimer (Fig. 4G) each resembled the P dimers of their HuNoV counterparts (Fig. 4E). Additionally, the open source software 3DLigandSite (65) indicated that the models retained ligand binding sites (red) for type A HBGAs (white spheres) similar to those seen with the GII.4 HuNoV backbone.

### BtCalV/A10 and BtNoV have different HBGA carbohydrate binding profiles, but both resemble HuNoV with respect to binding patterns.

**FIG 5:**
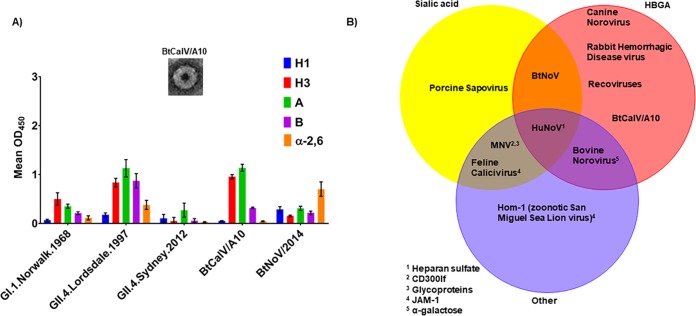
Bat caliciviruses retain HBGA binding patterns overlapping those of HuNoV VLPs. VLPs for BtCalV/A10 were generated using the VEE replicon in C6/36 cells. (A) The HBGA binding patterns of BtCalV/A10 and BtNoV were screened in comparison with those of HuNoVs (GI.1.Norwalk.1968, GII.4.1997.Lordsdale, and GII.4.Sydney.2012). The data presented are mean absorbance values (OD_450_) following subtraction of negative-control values ± SEM. Each VLP-HBGA interaction was measured in duplicate in two independent experiments. (B) Caliciviruses bind HBGAs, sialic acids, and other host ligands. Virus-ligand interactions were included in the Venn diagram for cases in which one publication had reported binding to the host ligand.

As we had shown that BtNoV VLPs could bind HBGAs (Fig. 2), we sought to identify whether the phylogenetically unrelated BtCalV/A10 shared similar host receptor ligand binding interactions. Thus, we utilized a synthetic carbohydrate binding assay to identify the potential HBGA receptor-binding patterns of BtCalV/A10 in comparison with a panel of VLPs derived from several HuNoVs (GI.1.Norwalk.1968, GII.4.Lordsdale.1997, and GII.4.Sydney.2012) and BtNoV (Fig. 5A).

Among the HuNoVs, GI.1.Norwalk.1968 VLPs primarily bound H type 3 and A antigens but weakly bound the H type 1 and B antigens and α-2,6-sialic acid. Conversely, GII.4.Lordsdale.1997 VLPs strongly bound H type 3, A, and B and α-2,6-sialic acid, with limited binding to H type 1. GII.4.Sydney.2012 VLPs displayed weak binding to all carbohydrates but bound most strongly to the A antigen. BtCalV/A10 VLPs bound strongly to A, B, and H type 3 HBGA carbohydrates but bound weakly to H type 1 and α-2,6-sialic acid. Of note, BtCalV/A10 VLPs bound strongly to the H type 3 and A HBGAs at a magnitude similar to that seen with the GII.4.Lordsdale.1997 HuNoV VLPs. Conversely, the BtNoV VLPs again bound to α-2,6-sialic acid but displayed modest binding to the H type 1 and H type 3, A, and B HBGA carbohydrates, as we showed previously (Fig. 2). Each VLP in our panel displayed limited or weak binding to H type 2 antigen, Lewis antigens, or α-2,3-sialic acid (data not shown). The bat caliciviruses exhibited different binding patterns: the BtCalV/A10 VLPs strongly bound H type 3, A, and B antigens, with limited binding to H type 1 and α-2,6-sialic acids, while the BtNoV VLPs strongly bound α-2,6-sialic acid, with moderate binding to H type 1 and H type 3, A, and B HBGAs, suggesting that bat caliciviruses can bind to a range of host receptors and offer multiple routes for cross-species transmission. Both of these binding patterns overlapped binding patterns that were previously reported for other animal caliciviruses and HuNoVs (Fig. 5B). Our results, coupled with those previous reports, suggest that multiple routes of cross-species transmission exist among caliciviruses.

## DISCUSSION

Bats are important reservoir hosts for newly emerging viruses, many with pandemic potential. The reservoir hosts for caliciviruses and noroviruses are presently unknown; the existence of distinct genera and genogroups, coupled with multiple variable genotypes, suggests that multiple novel independent introductions from animal populations may have contributed to the genetic diversity of noroviruses and caliciviruses. Here, we reveal the potential for several bat caliciviruses to transmit across multiple species. Specifically, we found that a previously reported but uncharacterized virus, BtNoV (50), is antigenically similar to HuNoVs and can bind HBGAs *in vitro*. We built upon this finding through identification of a bat calicivirus sequence (BtCalV/A10) from P. subflavus near an abandoned railroad tunnel outside Little Orleans, MD. We further characterized both bat caliciviruses through phylogenetic analyses, predictive structural homology modeling, VLP production, and identification of VLP-HBGA binding patterns *in vitro*. Our report suggests that BtCalV/A10 is most closely related to RHDV, a highly virulent calicivirus that emerged suddenly in China from preexisting strains prior to spreading globally (66, 67). While more sequences are needed for clarification, A10 likely represents a novel genus within *Caliciviridae*, while BtNoV likely belongs to GV with MNV. Structural homology modeling indicated that both bat viruses retain ligand binding sites similar to those seen with HuNoVs, validating their capability to bind HBGAs. The capsid sequences of both bat viruses formed VLPs similar to those formed by HuNoVs and showed overlapping HBGA binding profiles with respect to several HuNoVs and MNV. Importantly, as HBGAs are encoded across numerous animal species, particularly among mammals (68), similarities in attachment and entry cofactors suggest that these bat viruses have the potential to overcome a major roadblock to cross-species transmission across a variety of mammalian species.

Metagenomic sequencing has illuminated the great diversity and complexity of RNA viruses distributed throughout the natural world. However, new strategies are desperately needed to translate this information into meaningful predictions of biological processes, disease risk, and surveillance prioritization prior to epidemic or pandemic emergence. Previous studies on bat caliciviruses have focused on the use of phylogenetic analyses and structural modeling for classification of these novel viruses within the *Caliciviridae* family (45–49). While these techniques help contextualize viral relatedness within the framework of known and characterized viruses and their families, they do not necessarily aid in the understanding of biological functions or provide insight into the emergence potential of zoonotic pathogens. In this report, we utilized a novel platform combining metagenomics, phylogenetics, immunogenic comparisons, and homology modeling of two bat viruses; we showed that these viruses belong to *Caliciviridae* and are structurally similar to known human and animal caliciviruses. We coupled these data with *in vitro* techniques and immunologic assays to derive further relevant biological questions that can help to gauge the potential for cross-species transmission of two bat caliciviruses to other species prior to their emergence within the human population. A similar approach has identified the potential for MERS and SARS-like coronaviruses within Asian and African bat populations to replicate and emerge in human populations and to evade current therapeutics and has also identified bat coronaviruses that emerged more recently and caused outbreaks in swine (8, 9, 69, 70).

Specifically, we showed that BtNoV VLPs maintain similarity to HuNoV VLPs with respect to antigenic and receptor-binding patterns (Fig. 1 and 2). Additionally, BtNoV VLPs bound HBGA receptors across a variety of temperatures similarly to HuNoVs (Fig. 2), indicating the potential for viral transmission under a variety of physiological conditions. Further, we utilized a second, unrelated bat calicivirus to demonstrate that these observations may be pertinent across multiple bat calicivirus species. For example, BtCalV/A10 rooted a phylogenetic clade that included RHDV and "recoviruses," which were the only viruses separating BtCalV/A10 from noroviruses (Fig. 3), but likely represents a novel genus within the *Caliciviridae* family. Next, we modeled the P domain structure of BtCalV/A10 and BtNoV and host carbohydrate receptor binding sites on their capsid structure similar to those seen with known HuNoV structures (Fig. 4). Finally, we observed that BtCalV/A10 also formed VLPs of similar sizes and structures that bound to several HBGAs and/or sialic acid moieties and displayed binding patterns that overlapped those of HuNoVs *in vitro* (Fig. 5A and B). However, despite the similarity in the predicted P domain structures, the bat viruses, similarly to GI and GII HuNoVs, displayed disparate HBGA binding patterns, indicating multiple potential paths for cross-species transmission (Fig. 5B). Therefore, the utilization of the aforementioned platform identified antigenic relatedness of BtNoV and HuNoVs as well as the potential for cross-species transmission of two previously uncharacterized or unidentified bat caliciviruses.

Noroviruses have been historically classified on the basis of capsid sequence homology (59); based on this strategy, BtNoV would likely be classified within *Norovirus* GV alongside MNV. Enzyme immunoassays have been powerful tools for understanding antigenic relationships among noroviruses in spite of sequence homology, especially in elucidating how antigenic drift contributes to the evasion of herd immunity and emergence of novel HuNoV strains (35). In the present study, EIA performed with polyclonal serums derived from historic and current strains of HuNoVs showed that BtNoV VLPs are antigenically similar (Fig. 1) despite the parental virus’s phylogenetic relatedness to MNV (Fig. 3). Other bat noroviruses have shown extensive homology with GIV noroviruses (51), whose antibodies have been detected in humans and canine species (71, 72). These results further illustrate the importance of our proposed platform: capsid sequence homology alone would not have predicted that bat caliciviruses could bind synthetic carbohydrates or be detected by serums against human viruses; *in vitro* assays revealed novel information regarding the caliciviruses circulating in bat populations.

The *FUT2* gene, whose protein product promotes expression of HBGAs on mucosal surfaces, has a well-established role in HuNoV infection and RHDV disease (73–75). However, the receptors for animal caliciviruses are variable and less clear. For instance, GIII bovine noroviruses bind α-galactose (76, 77), MNVs bind sialic acids and CD300lf (78–80), porcine sapoviruses bind sialic acids (81), and both feline calicivirus and the zoonotic San Miguel sea lion virus Hom-1 bind junctional adhesion molecule-1 (82, 83). While protein receptors seem quite variable, there is significant overlap in the identified carbohydrate binding patterns of all caliciviruses (Fig. 5), demonstrating multiple potential routes of cross-species transmission of these viruses.

Recent genomics analysis has further indicated the potential for animal caliciviruses, including bat caliciviruses, to cross species barriers. For instance, HBGAs have been identified or predicted across numerous animal species (68). Importantly, the A and B fucosyltransferase enzymes of Myotis lucifugus, a microbat similar to P. subflavus, contain amino acid sequences, amino acid motifs, and levels of phylogenetic relatedness similar to those of their human orthologs (68). Similarly, CD300lf orthologs have been predicted in numerous animal species, including several Asian bats, such as Hipposideros armiger and Rhinolophus sinicus. In addition to CD300lf, sialic acids have previously been implicated as receptors or cellular cofactors for binding and entry by MNVs and, more recently, by HuNoVs (78, 84–86). Future studies will reveal whether the BtCalV/A10 or BtNoV VLPs also bind bat or mammalian CD300lf proteins. Though numerous factors mediate the cross-species transmission, replication, and pathogenesis of viruses, our results, coupled with those revealing the analogous host receptor structures, suggest that these bat caliciviruses have the potential to overcome host receptor barriers as a roadblock to cross-species transmission.

Our findings, along with previous reports (45, 46, 48–50), support the hypothesis that bats are a potential reservoir species for noroviruses and other caliciviruses, such as RHDV. While the BtCalV/A10 strain is very distant from RHDV, our data strongly suggest that more bat caliciviruses should be identified and sequenced, primarily in China, and that the results may reveal precursor strains that are more akin to this highly virulent pathogen of rabbits. The present study did not support the conclusion that bat caliciviruses will emerge in human or other mammalian populations but did suggest their potential for cross-species transmission. Our results further illustrate the importance of conducting continued surveillance of bat populations for viral pathogens prior to their emergence in human and other mammalian populations (11). If viral discovery is partnered with developing and answering meaningful biological questions to identify high-risk viral strains prior to epidemic or pandemic emergence, the cost of such combined scientific investigations will pale in comparison to the cost of treatment and eradication of these pathogens as they emerge in the human population.

## MATERIALS AND METHODS

### Viral genomic sequence isolation and identification.

Viral genomic sequences were identified as part of a previous study (52). Briefly, bats were collected with harp traps at an abandoned railroad tunnel near Little Orleans, MD, and fresh guano was collected. Samples were stored on ice until processing. The BtCalV/A10 capsid sequence was isolated from Perimyotis subflavus (tri-colored bat) using the sequence-independent single-primer amplification technique and 454 next-generation sequencing platform. *De novo* contigs were assembled using three programs: Codon Code Aligner, Geneious, and DNAStar. Viral amino acid sequences were created using Vector NTI. Assembled contigs were analyzed using the basic local alignment search tool (BLAST) from the National Center for Biotechnology Information (NCBI) (87). BLAST searches were conducted at the amino acid level using the protein-protein BLAST (blastp) function to query nonredundant protein sequences.

### Phylogenetic analysis.

Maximum-likelihood cladograms of 50 known calicivirus VP1 amino acid sequences and 51 known calicivirus S domain amino acid sequences were generated using MEGA7. The cladograms were generated based on the JTT matrix-based amino acid substitution model and initially derived with the Neighbor-Join (NJ) and BioNJ algorithms followed by pairwise distance estimation by the use of the JTT amino acid replacement model. Bootstrapping was conducted by generating 500 bootstrapped replicates, and a consensus tree was developed using MEGA7. Consensus radial phylograms were generated in Geneious R11, employing the same sequences used to construct the cladograms, with the Jukes-Cantor genetic distance model, the NJ build method, no outgroup, and 100 bootstrap replicates. Phylograms were rendered for publication in Adobe Illustrator CC 2017.

### Homology modeling.

The full-length BtCalV/A10, GII.4.1997.Lordsdale HuNoV, GI.1 human sapovirus, GV.1 MNV, and BtNoV capsid sequences (50) were submitted to the Protein Homology/analogy Recognition Engine V2.0 (PHYRE2) server (88) and analyzed using Intensive Mode. Homology modeling of the BtCalV/A10 and BtNoV sequences was based on the known HuNoV GII.4/VA387 (PDB ID: 2OBT) (89) and HuNoV GII.4.2012/NSW0514 bound to type A trisaccharide (PDB ID: 4WZT) (90) and MNV (PDB ID: 3LQE) structures from the Protein Data Bank. The amino acid sequences were aligned using the Clustal Omega platform on the Bioinformatics Toolkit from the Max Planck Institute (62, 91). The predicted tertiary structure of the aligned sequence was determined by the use of the Modeller platform from the Bioinformatics Toolkit from the Max Planck Institute using GII.4.2012/NSW0514 as the template (63). Dimeric models were created using The PyMOL Molecular Graphics System, version 1.7.4.3 (Schrödinger, LLC).

### Generation of virus-like particles.

Virus-like particles (VLPs) were generated as previously described (92). Briefly, the BtCalV/A10 and BtNoV VP1 sequences were synthesized and inserted directly into the VEE 3526 replicon. VP1 mRNAs were derived via T7 polymerase *in vitro* transcription. The VEE-BtCalV/A10 and VEE-BtNoV mRNAs were separately electroporated in C6/36 mosquito larva (Aedes albopictus) cells (ATCC) and BHK cells, respectively. Electroporated C6/36 and BHK cells were incubated at 32°C and 37°C, respectively, with 5% CO_2_ for 26 to 28 h before harvesting VLPs. VLPs were purified by velocity sedimentation in sucrose and concentrated in phosphate-buffered saline (PBS) using 100-molecular-weight-cutoff (MWCO) centrifugal filter units (Millipore). VLP formation and structure were confirmed by transmission electron microscopy. Protein concentrations were determined by the use of the bicinchoninic acid (BCA) protein assay (Pierce). VLPs for GI.1.Norwalk.1968, GII.4.1997.Lordsdale, and GII.4.Sydney.2012 HuNoVs and MNV were previously synthesized and stored at −80°C until use.

### Carbohydrate binding assays.

A carbohydrate binding assay to detect VLP binding was conducted as previously described (93, 94). Briefly, Costar enzyme immunoassay (EIA) plates (Corning) were coated with 2 µg/ml of each of the produced VLPs mixed with PBS and incubated for 4 h at room temperature. Plates were blocked overnight with blotto (5% instant dry milk–PBS–Tween 20). Synthetic biotinylated carbohydrates (Glycotech) diluted in blotto (10 µg/ml) were added to each well and incubated at 37°C for 1 h. For the temperature-dependent HBGA binding assay, the plates were incubated at 25°C, 32°C, and 37°C for 1 h. Streptavidin-horseradish peroxidase (HRP) was diluted in blotto (1:10,000) and incubated at 37°C for 30 min. The plates were developed with One-Step Ultra-TMB (Thermo Fisher) for 15 min and were stopped with 2 M H_2_SO_4_ prior to being read at 450 nm. Plates were washed 3 times with PBS–0.05% Tween between steps. The following 11 carbohydrates were analyzed: H type 1, H type 2, H type 3, A, B, Lewis A, Lewis B, Lexis X, Lewis Y, α-2,3-sialic acid, and α-2,6-sialic acid. PBS served as a negative control for VLP coating, and blotto served as a negative control for HBGA binding. Each HBGA-VLP interaction was evaluated in duplicate in at least two independent experiments. Data are presented as mean absorbance values ± standard errors of the means (SEM) at 450 nm after removal of PBS negative-control absorbance values.

### Enzyme immunoassay.

An enzyme immunoassay (EIA) to determine the antigenic relationships of VLPs was conducted as previously described (53, 95). Briefly, Costar EIA plates (Corning) were coated with 2 µg/ml of VLPs from BtNoV, GI.1.Norwalk.1968, GII.4.Lordsdale.1997, GII.4.Sydney.2012, and MNV mixed with PBS for 4 h at room temperature and blocked overnight with blotto (5% instant dry milk–PBS–Tween 20). Rabbit hyperimmune serum against GI and GII.4 HuNoVs was diluted 1:2 in blotto, added to each plate, and incubated for 1 h at 37°C. HRP-conjugated anti-rabbit secondary antibodies were diluted in blotto, added to each plate, and incubated for 30 min at 37°C. Negative controls included wells with no VLP and no primary antibody. Plates were developed with One-Step Ultra-TMB (Thermo Fisher) and 2 M H_2_SO_4_ and read immediately at 450 nm. Plates were washed 3 times with PBS–0.05% Tween between steps. Data are presented as mean absorbance ± SEM at 450 nm after removal of negative-control absorbance. To determine EC_50_, EIA data were log transformed and fitted using sigmoidal dose-response analysis of nonlinear data in GraphPad Prism 7.02 (GraphPad Software, Inc., La Jolla, CA). EC_50_s were compared using one-way analysis of variance (ANOVA) with Dunnett’s posttest.
