# Supplementary figures and images for: Bat Caliciviruses and Human Noroviruses Are Antigenically Similar and Have Overlapping Histo-Blood Group Antigen Binding Profiles
Source: mBio. 2018 May 22;9(3):e00869-18. doi: 10.1128/mBio.00869-18 (PMC5964351; doi:10.1128/mBio.00869-18)

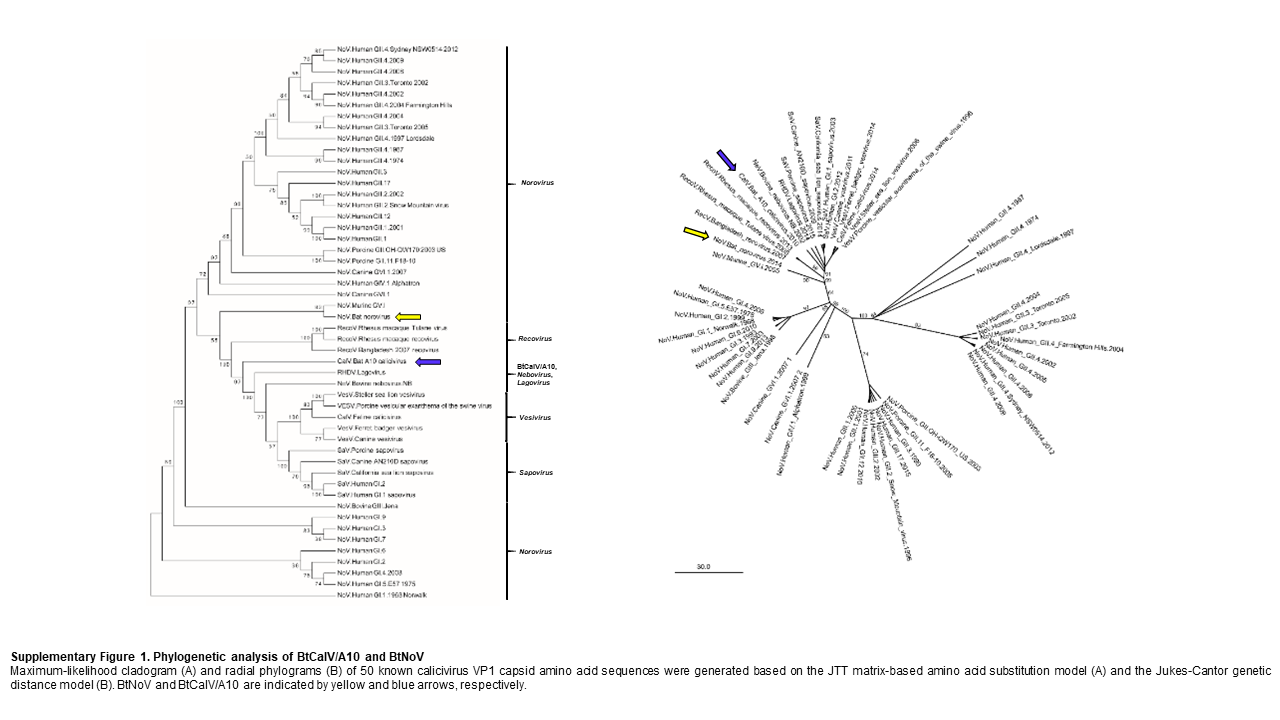

Supplement: FIG S1 [file mbo003183900sf1.tif]
