# Supplementary material for: Bat Caliciviruses and Human Noroviruses Are Antigenically Similar and Have Overlapping Histo-Blood Group Antigen Binding Profiles
Source: mBio. 2018 May 22;9(3):e00869-18. doi: 10.1128/mBio.00869-18 (PMC5964351; doi:10.1128/mBio.00869-18)
Supplement: TABLE S1 [file mbo003183900st1.docx]

|  |  | **VP1** | | **S domain** | |
| --- | --- | --- | --- | --- | --- |
|  | **Genus** | **Consensus** | **Identity** | **Consensus** | **Identity** |
| **NoV.Human_GI.1.1968_Norwalk** | ***Norovirus*** | 32.1 | 16.6 | 37.0 | 20.2 |
| **NoV.Human_GII.4.Sydney_NSW0514_2012** | ***Norovirus*** | 31.9 | 18.2 | 38.8 | 23.6 |
| **NoV.Human_GII.4.1997_Lordsdale** | ***Norovirus*** | 32.2 | 19.2 | 42.7 | 24.8 |
| **NoV.Murine_GV.I** | ***Norovirus*** | 31.3 | 15.8 | 40.0 | 21.7 |
| **CalV.Bat_M63_HUN_2013** | **Unclassified** | 32.3 | 17.0 | 44.1 | 25.0 |
| **CalV.Bat_BS58_HUN_2013** | **Unclassified** | 30.5 | 16.1 | 40.8 | 21.4 |
| **RecoV.Rhesus_macaque_Tulane virus** | ***Recovirus*** | 32.5 | 17.3 | 38.6 | 21.6 |
| **RecoV.Bangladesh_2007_recovirus** | ***Recovirus*** | 28.2 | 13.9 | 35.1 | 16.7 |
| **VesV.Feline_calicivirus** | ***Vesivirus*** | 30.6 | 15.5 | 39.8 | 22.5 |
| **LagV.RHDV** | ***Lagovirus*** | 31.2 | 19.2 | 39.6 | 24.9 |
| **NeV.Bovine_nebovirus.NB** | ***Nebovirus*** | 30.7 | 16.4 | 41.3 | 21.5 |
| **NoV.Bat_norovirus** | ***Norovirus*** | 31.9 | 17.0 | 40.2 | 22.8 |
| **SaV.Bat_TLC58_HK** | ***Sapovirus*** | 28.6 | 15.6 | 38.2 | 22.4 |
| **SaV.Human_GI.1_sapovirus** | ***Sapovirus*** | 31.1 | 16.1 | 41.5 | 23.3 |

**Supplementary Table 1. Amino acid sequence similarity between BtCalV/A10/USA/2009 and caliciviruses**

**Supplementary Table 1. Sequence similarity between BtCalV/A10 VP1 and S domain amino acid sequences relative to other caliciviruses.** The BtCalV/A10 VP1 and S domain sequences were aligned in VectorNTI and are presented as percent consensus amino acid and sequence identity.
